# Prevalence, mortality, and aetiology of paediatric shock in a tertiary hospital in Malawi: A cohort study

**DOI:** 10.1371/journal.pgph.0002282

**Published:** 2024-01-08

**Authors:** Mercy Kumwenda, Roxanne Assies, Gloria Chathima, Harriet Khofi, Job B. M. van Woensel, Yamikani Chimalizeni, Josephine Langton, Job C. J. Calis

**Affiliations:** 1 Department of Paediatrics and Child Health, Kamuzu University of Health Sciences, Blantyre, Malawi; 2 Department of Paediatrics and Child Health, Kamuzu Central Hospital, Lilongwe, Malawi; 3 PICU, Emma Children’s Hospital, Amsterdam UMC Location University of Amsterdam, Amsterdam, The Netherlands; 4 Amsterdam Reproduction & Development, Amsterdam, The Netherlands; University of California San Francisco, UNITED STATES

## Abstract

Shock is considered one of the most important mechanisms of critical illness in children. However, data on paediatric shock in sub-Saharan Africa is limited, which constrains development of effective treatment strategies. We aimed to describe the prevalence, mortality, and aetiology of paediatric shock in a tertiary hospital in Malawi. Children aged two months to 16 years presenting with shock (FEAST criteria; respiratory distress and/or impaired consciousness, and at least one sign of impaired circulation; capillary refill>3 seconds, cold extremities, weak pulse, or severe tachycardia) to the emergency department were included and followed-up prospectively using routinely collected data between February 2019 and January 2020. Prevalence, mortality and aetiology of shock were reported for both the FEAST criteria and World Health Organization (WHO) definition. The association between aetiology and mortality was assessed with univariable analysis. Of all screened admissions (N = 12,840), 679 (5.3%) children presented with shock using FEAST criteria and the mortality was 79/663 (11.9%). WHO-defined shock applied to 16/12,840 (0.1%) and the mortality was 9/15 (60.0%). Main diagnoses were viral/reactive airway diseases (40.4%), severe pneumonia (14.3%), gastroenteritis (11.3%) and presumed sepsis (5.7%). Children diagnosed with presumed sepsis and gastroenteritis had the highest odds of dying (OR 11.3; 95%-CI:4.9–25.8 and OR 4.4; 95%-CI:2.4–8.2). Considering the high mortality, prevalence of paediatric shock (FEAST and WHO definitions) in Malawi is high. Sepsis and gastroenteritis are diagnoses associated with poor outcome in these children. Consensus on a clinical meaningful definition for paediatric shock is essential to boost future studies.

## Introduction

Globally, 5 million deaths of under-five year old children occurred during 2020, of which more than half occurred in sub-Saharan Africa [1]. In-hospital mortality in these countries remains high and most of these deaths occur within the first 24 hours of admission [2]. Improving emergency care is essential to increase survival of these critically ill children.

The World Health Organization (WHO) Emergency Triage Assessment and Treatment (ETAT) provides guidelines for emergency care of critically ill children in low-resource settings, focussing on respiratory distress, circulatory failure and seizures [3]. Treatment of circulatory failure, or shock, in children in sub-Saharan Africa, however, has been an issue of debate since the largest trial on fluid bolus treatment in paediatric shock was published in 2011 (the Fluid Expansion As Supportive Therapy or FEAST trial). This trial showed that administration of fluid boluses could have detrimental effects in children with febrile illness and impaired perfusion [4]. The WHO has subsequently adapted the ETAT guidelines to include these results, distinguishing between different definitions for shock and treatment algorithms [5].

The publication of the results of the FEAST trial initiated a still unresolved debate regarding the optimal treatment for shock in children in sub-Saharan Africa and other LMIC. Since then, at the time of writing, only one study by Mbevi et al. has been published focusing on shock in African children [6]. Furthermore, the lack of a universal definition for shock hampers the interpretation of study results. The FEAST trial applied criteria based on international guidelines that are different from the more stringent definition for paediatric shock in the WHO ETAT guideline [4, 5]. These definitions mostly consist of combinations of clinical signs, including capillary refill time, cold peripheries, heart rate and blood pressure, which also has its limitations. For example, increased heart may also be seen in several other conditions in which circulation is not necessary compromised such as fever, severe respiratory distress, or pain. Few studies have reported these different definitions and subsequent differences in interpretation of study results in a low-resource setting other than the ones included in the FEAST trial [6, 7].

Without improved understanding of the impact of the FEAST trial and other shock definitions on the prevalence, outcome and aetiology of shock it is difficult to design adequate treatment protocols for paediatric shock in general and specifically for our context. Therefore, we performed this cohort study to assess the prevalence, mortality and potential aetiology of shock in children in Malawi, applying both the FEAST and WHO definition. First, we aimed to determine the prevalence of shock in children upon admission to hospital and their clinical outcome. Second, we aimed to describe clinical characteristics and aetiology of children with shock, and the association between aetiology and mortality.

## Methods

### Study setting

This study was conducted at Queen Elizabeth Central Hospital (QECH), a large tertiary hospital in Blantyre, Malawi, that has a catchment area of over six million people, of whom more than half are children under 15 years old [8, 9]. All children are admitted through the paediatric accident and emergency department to one of the five paediatric wards. Local guidelines for shock follow recommendations in WHO ETAT and clinicians at QECH are trained in WHO ETAT guidelines [3]. Reporting was done using the STROBE recommendations [10].

### Study design and participants

**Fig 1:**
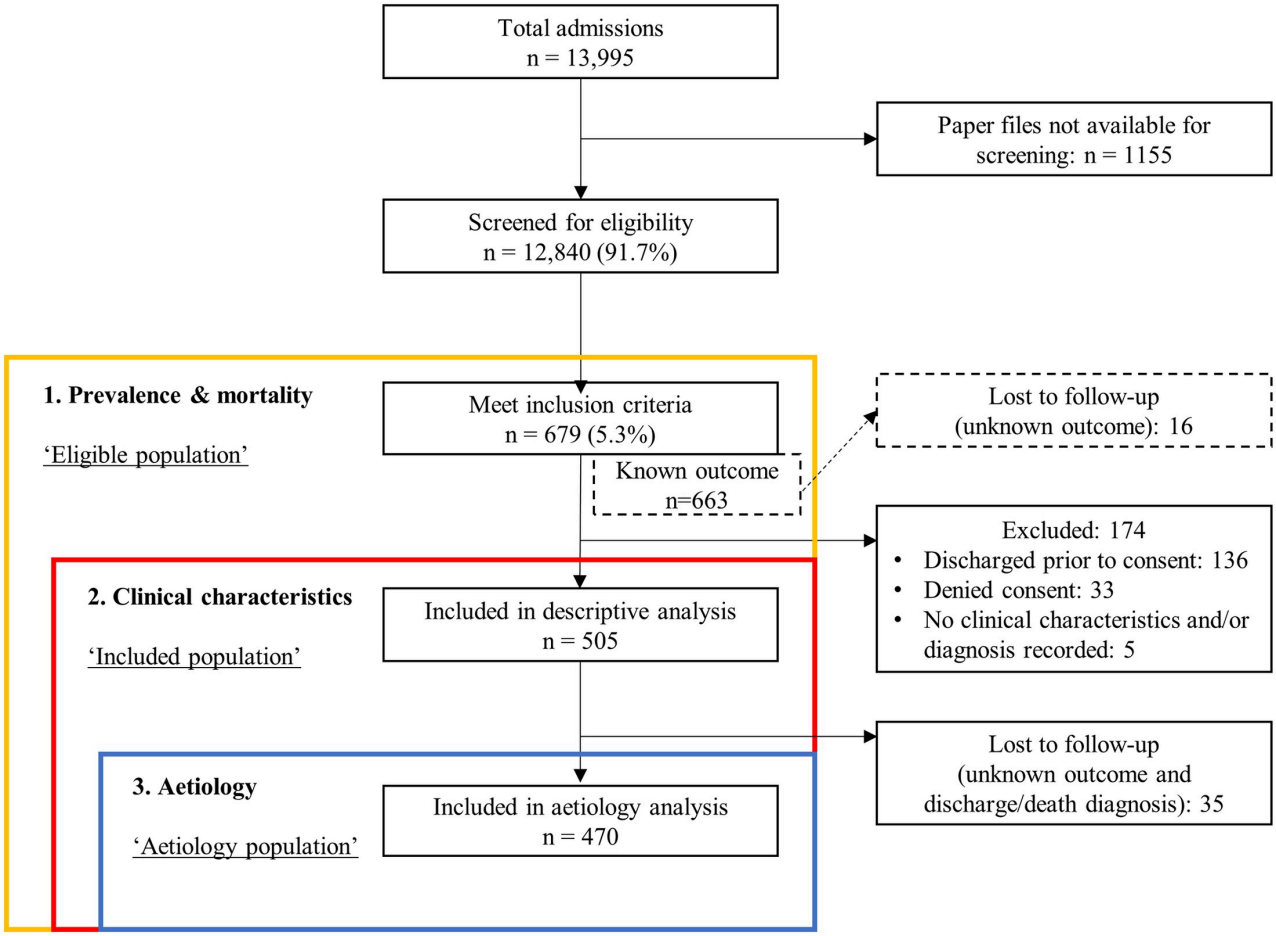
Flow diagram of included children for different analysis stages. The ‘eligible population’ consists of all children that meet the inclusion criteria. The ‘included population’ are children for whom consent was given for analysis of clinical characteristics and follow-up of these children. The ‘aetiology population’ consists of all children with known outcome and discharge/death diagnosis.

Children aged two months to 16 years with shock upon admission were included and followed up prospectively until discharge or death using routinely collected data over a 12-month period (1 February 2019–31 January 2020). Shock was defined using a modified FEAST definition, to be able to compare our results with the FEAST trial, the landmark paper on shock in African children (S1 Table). The definition is modified by applying it to all children presenting to the emergency department, not only to children presenting with febrile illness. All paper hospital admission files were screened daily. For eligible children, informed consent was subsequently obtained from parents or guardians. Therefore, two study populations were considered, an eligible population consisting of children that met the inclusion criteria and an included population consisting of children whose guardian or parents consented to use further clinical details for this study. For children who died before parents or guardians could give informed consent, we received ethical approval to include them for further analysis in this study. By including these children, we attempted to avoid selection bias in further assessment of the aetiology of shock. Children were prospectively followed up by retrieving their paper admission files daily during weekdays or the next working day if they were admitted on weekends and public holidays. Data were manually entered from the admission file into an electronic case record form in Open Data Kit (2020 Get ODK Inc.) after discharge or death. Fig 1 shows a flow diagram of screened, eligible and included children.

### Data collection

The primary outcome was in-hospital mortality. Prevalence and mortality were derived from the eligible population (all children who met the inclusion criteria), for which the total screened children was used as the denominator to represent all admissions through the emergency department. None of the eligible children were discharged directly home or transferred to another hospital. A subset of the eligible population also classified as WHO shock, or as “shocked” by the clinician (S1 Table) [5]. If one of these variables was missing, it was considered normal. The paediatric department uses a predefined and concise paper hospital admission form that is completed by the attending physician in the emergency department, which we used for the case record form (S1 Fig). Vital signs are therefore the first upon presentation before initial treatment, recorded by the clinician. All additional tests were independently done as indicated by the clinician or routinely as part of the services offered at QECH (S2 Table). Aetiological diagnosis was the clinical diagnosis recorded by the attending clinician upon discharge. To assess the aetiology of shock, we grouped the main discharge diagnosis of the included children into diagnostic categories, which were considered as exposure variables (S3 Table).

### Missing data

Patient outcome was extracted from hospital logbooks and additional information on the discharge or death diagnosis was retrieved from the paper admissions files. Lost to follow-up of outcome and/or main discharge diagnosis was reported. Main reasons for missing data were not recorded clinical data by attending physicians or missing paper files.

### Statistical analysis

Data were analysed using IBM SPSS version 23. The prevalence of shock was calculated by dividing the total number of eligible children with shock on admission by the total number of screened paediatric admissions. Mortality was calculated by dividing the total number of deaths by the total eligible children with known outcome. Diagnostic categories were compared between the two definitions for shock using Chi square or Fisher’s exact test for categorical data. A p-value of <0.05 was considered statistically significant. In an explorative analysis we assessed associations between diagnosis and mortality using univariable analysis and presenting odds ratios and 95% confidence intervals.

### Ethics statement

For this observational study, we obtained required approval by the College of Medicine Research and Ethics Committee (COMREC –P.12/18/2557). Parents or guardians of eligible children provided written informed consent. The ethical committee provided a waiver to include the data of children who died prior to consent. During data collection, MK, RA and GC had access to individual patient information. The dataset includes anonymized data. Additional information regarding the ethical, cultural, and scientific considerations specific to inclusivity in global research is included in the S1 Checklist.

## Results

Of the 13,995 admitted children during the 12 months, 12,840 (91.7%) hospital files could be retrieved for screening. Of these 12,840 children, 679 fulfilled the eligibility criteria for shock based on the FEAST criteria (eligible population) from which prevalence and mortality could be derived. Of these children, 505 could be included for descriptive analysis of clinical characteristics and 470 children for further analysis of aetiology and mortality (Fig 1).

### Prevalence

Prevalence of shock on admission amongst all screened children was 5.3% (679/12,840, Fig 1). The WHO shock definition applied to 0.1% (16/12,840) of all screened children. Clinicians reported shock as an admission diagnosis in 0.3% (36/12,840) of all screened children.

### Outcome (mortality)

Outcome was known for 663 of 679 (97.6%) children with shock. Of these 663 children, 79 (11.9%) died. For WHO shock, outcome was known for 15 of 16 children. Of these 15 children, nine (60%) children died.

### Clinical characteristics at baseline

Of the 505 children included for further analysis, median age was 17.0 (9.0–36.0) months and 56.6% were male. Fever was reported as a presenting symptom in 77.4% of the children; other main presenting symptoms were respiratory distress (81.4%) and cough (75.1%). On examination, the most frequent clinical signs were severe tachycardia (89.6%) and respiratory distress (82.5%). Fever (>39°C) was measured in 18.0%, pallor in 19.9%, and signs of dehydration in 14.9%. In children with WHO shock, the main presenting symptoms were fever (73.3%) and vomiting (73.3%). The main clinical signs in these children were prostration (93.3%), coma (86.7%), pallor (53.3%), and signs of dehydration (53.3%). Blood cultures were taken on clinical indication in 176/505 (34.9%) children and 8/176 (4.5%) were positive for a micro-organism. Death was more common early during admission, with 27.6% occurring within 24 hours and 55.3% occurring within 48 hours. Table 1 describes clinical characteristics, laboratory results, and management.

**Table 1:** Clinical characteristics of included children (’included population’) for two definitions for shock: FEAST criteria and WHO shock definition.

	FEAST criteria (N = 505)^a^	WHO criteria (N = 15)^a^
**Demographics**		
Age in months (Median [IQR])	17.0 (9.0–36.0)	11.0 (7.0–25.0)
Sex (male)	282/498 (56.6)	11/15 (73.3)
Completed vaccinations	264/439 (60.1)	4/11 (36.4)
Underlying conditions	71/456 (15.6)	3/12 (25)
**Presenting symptoms (reported by guardian)**		
Respiratory distress	397/488 (81.4)	8/15 (53.3)
Fever	383/495 (77.4)	11/15 (73.3)
Cough	364/485 (75.1)	4/14 (28.6)
Vomiting	183/484 (37.8)	11/15 (73.3)
Difficulty in feeding	181/462 (39.2)	11/15 (73.3)
Diarrhoea	127/478 (26.6)	9/15 (60.0)
Convulsions	69/476 (14.5)	4/15 (26.7)
Pallor	43/457 (9.4)	2/14 (14.2)
Rash	26/458 (5.7)	3/14 (21.3)
Oedema	19/463 (4.1)	1/14 (7.1)
Jaundice	10/462 (2.2)	1/14 (7.1)
Trauma	3/491 (0.6)	0/15 (-)
**Physical exam in the emergency department**		
Temperature^b^ >39°C (fever)	86/477 (18.0)	3/14 (21.4)
Temperature <36°C (hypothermia)	24/477 (5.0)	2/14 (14.3)
Respiratory distress (increased work of breathing)	416/504 (82.5)	11/15 (73.3)
Prostration/lethargy	146/482 (30.3)	14/15 (93.3)
Coma (BCS ≤4)	119/485 (24.5)	13/15 (86.7)
Severe tachycardia^c^	441/492 (89.6)	12/15 (80.0)
Weak radial pulse	47/91 (51.6)	15/15 (100)
CRT >3 seconds	71/457 (15.5)	15/15 (100)
Cold peripheries	78/136 (57.4)	15/15(100)
Dehydration^d^	72/484 (14.9)	8/15 (53.3)
Pallor	97/488 (19.9)	8/15 (53.3)
Jaundice	15/488 (3.1)	1/15 (6.7)
Oedema	18/490 (3.7)	0/15 (-)
Cardiac signs (such as murmur, yes/no)	25/491 (5.1)	0/14 (0-)
Poor nutrition^e^	39/471 (8.3)	4/15 (26.7)
**Laboratory Results**		
HIV-infected	27/358 (7.5)	2/13 (15.3)
HIV-exposed^f^	31/358 (8.7)	5/13 (38.4)
RBS^g^ <2.4 or <3mmol/L	25/272 (9.2)	6/15 (40.0)
RBS > 10mmol/L	45/272 (16.5)	2/15 (13.3)
Hb ≤5g/dL	19/334 (5.7)	1/13 (7.7)
Hb >5–< 10g/dL	129/334 (38.6)	6/13 (46.2)
Positive malaria test	67/395 (17)	4/14 (28.6)
Positive blood culture^h^	8/176 (4.5)	0/8 (-)
**Management in the emergency department**		
Fluid bolus	83/505 (16.4)	13/15 (86.7)
Blood transfusion	48/505 (9.5)	5/15 (33.3)
Antibiotics	231/505 (45.7)	11/15 (73.3)
Antimalarials	77/505 (15.2)	4/15 (26.7)

^a^ Denominator in each row indicating for how many children this variable was recorded.

^b^ Axillary temperature measurement

^c^ Severe tachycardia defined as >180 beats per minute if < 12 months of age, > 160 beats per minute if one to five years, > 140 beats per minute if five to 12 years and >120 beats per minute if 12 to 16 years of age

^d^ Clinician’s assessment: lethargy, sunken eyes, delayed skin pinch

^e^ Eyeball assessment of admitting clinician

^f^ Children <1 year of age with an HIV positive mother but no confirmed HIV infection in the child

^g^ Random blood sugar

^h^ Positive blood culture results: 2 Staphylococcus Aureus, 1 Salmonella typhimurium, 1 Salmonella, 1 Methicillin resistant Staphylococcus Aureus, 1 Klebsiella, 2 Escherichia Coli, 1 Acinetobacter Baumanii.

### Aetiology

**Fig 2:**
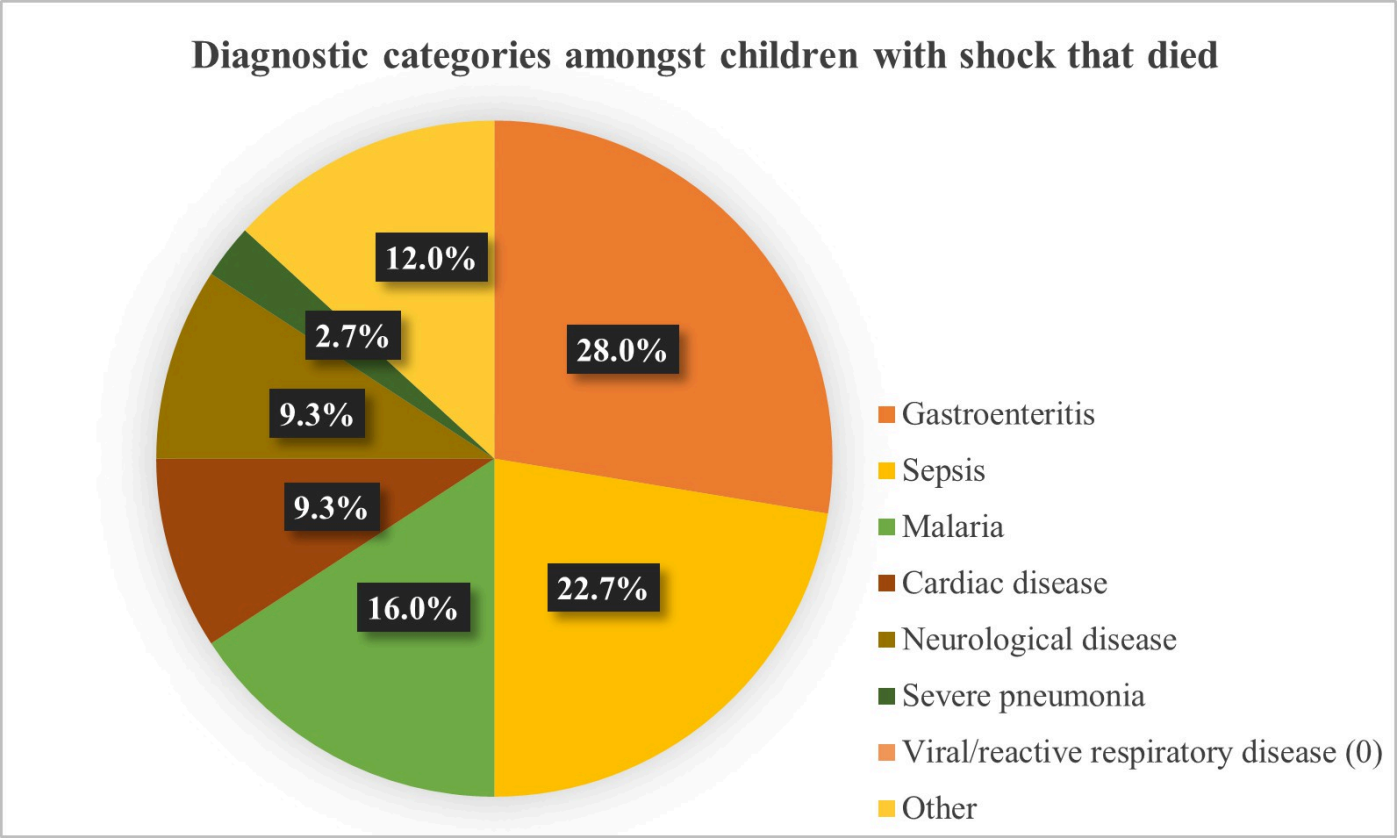
Diagnostic categories amongst children who died. (N = 75).

Of the 505 included children, 470 (93.1%) children had outcome and diagnosis recorded. Two or more diagnoses were recorded in 190/470 (40.4%). The predominant diagnostic category was viral/reactive respiratory diseases (40.4%). Severe pneumonia was recorded as the main diagnostic category in 14.3%, gastroenteritis in 11.3%, malaria in 12.6%, and sepsis in 5.7% of children with shock according to the FEAST criteria. In children with WHO shock, the main diagnostic categories were gastroenteritis (40.0%), sepsis (26.7%) and malaria (26.7%) (Table 2). In the children that died (n = 75), gastroenteritis (28.0%) and sepsis (22.7%) were the most common diagnoses (Fig 2, S4 Table). Children with HIV had slight increased odds of dying, although this was not statistically significant, while children with poor nutrition had increased odds of dying (OR 1.26; 95%-CI: 0.97–1.63 and OR 1.85 95%-CI: 1.29–2.66, respectively). Children diagnosed with sepsis and gastroenteritis had the highest odds of dying (OR 11.3; 95%-CI: 4.9–25.8 and OR 4.4; 95%-CI: 2.4–8.2, respectively) (Table 2).

**Table 2:** Diagnostic categories in the aetiology population (N = 470), when applying the WHO definition (N = 15), as well as explorative analysis assessing associations between diagnostic category and mortality. Diagnostic categories where based on the main discharge diagnosis by the clinician.

Diagnostic category	FEAST criteria (N = 470)	WHO shock (N = 15)	Mortality in children with diagnosis (%)	Mortality in children without diagnosis (%)	Odds Ratio (95% CI)
	n/N (%)	n/N(%)			
Viral/reactive respiratory disease	190 (40.4)	0 (-)	0/190 (-)	75/279 (26.9)	0.01 (0.00–0.12)
Severe pneumonia	67 (14.3)	0 (-)	2/67 (3.0)	73/403 (18.1)	0.14 (0.03–0.58)
Gastroenteritis	53 (11.3)	6 (40.0)	21/53 (39.6)	54/417 (12.9)	4.41 (2.37–8.20)
Sepsis	27 (5.7)	4 (26.7)	17/27 (63.0)	58/443 (13.1)	11.3 (4.93–25.8)
Malaria	59 (12.6)	4 (26.7)	12/59 (20.3)	63/411 (15.3)	1.41 (0.71–2.81)
Neurological disease	25 (5.3)	0 (-)	7/25 (28.0)	68/445 (15.3)	2.16 (0.87–5.36)
Cardiac disease	19 (4.0)	0 (-)	7/19 (36.8)	68/451 (15.1)	3.29 (1.25–8.64)
Other	30 (6.4)	1 (6.7)	9/30 (30.0)	66/440 (15.0)	2.43 (1.07–5.53)

## Discussion

Prevalence of paediatric shock in this cohort study was 5.3% applying the FEAST criteria in children on admission to a tertiary hospital in Malawi. Mortality was 11.9%. Both numbers were highly dependent on the definitions for shock used as prevalence was lower and mortality higher when applying the WHO definition. Aetiologies associated with increased mortality included sepsis and gastroenteritis. Respiratory diseases including bronchiolitis, asthma, and severe pneumonia were most commonly reported as the main underlying diagnosis in shock according to FEAST criteria, however had a low mortality.

### Prevalence, mortality and definitions

A uniform bedside definition for paediatric shock that can be used in low-resource settings is lacking [5, 7]. In this study, we reported prevalence and mortality for two definitions for shock. We primarily chose the FEAST criteria for this study as it was used in the largest trial on paediatric shock and its treatment in sub-Saharan Africa to date, and found that it applied to 5.3% of children admitted to QECH. Although the FEAST trial is an important study, neither FEAST nor other studies reported prevalence of shock using the FEAST criteria for shock. Secondly, we applied the WHO shock definition, as it is the most important triage and treatment guideline for low-resource settings worldwide, and found a prevalence of 0.1% of all paediatric admissions. This prevalence is in line with other studies in African children, and corroborates with the FEAST trial reporting that only two percent of these children had WHO defined shock [6, 7].

The different definitions also affected the mortality of shock in this study, which was 11.9% applying the FEAST criteria. This was slightly higher than in the FEAST trial, that reported a 48 hour mortality ranging between 7.3–10.5% [4], which may be explained by better outcomes which are common in randomized controlled trials. We found that mortality in children with WHO defined shock was much higher (60%). This is in line with mortality reported in other studies for children with WHO defined shock, ranging from 41.5% to 100% [7, 11].

Based on the different prevalence and mortality, we believe that neither the FEAST nor the WHO criteria accurately describe paediatric shock. The FEAST criteria may result in overreporting of shock, as nearly half of the children had reactive airway disease which was associated with a mortality of 0%. The WHO definition may result in an underestimation of shock prevalence as it appears to describe children with an advanced stage of shock. These children had extremely high mortality and clinicians reported shock as an admission diagnosis in at least twice the number of children, a concern also raised by other researchers [6, 7]. However, the WHO ETAT guideline and other researchers suggest that the presence of some, but not all, clinical signs of shock used in the WHO definition may be less specific for shock and could also be due to other conditions such as fever, hypoxemia, pain, or exposure [5, 12, 13]. Irrespective of the exact prevalence and mortality, shock is an important clinical syndrome in African children as it has a high mortality and given its severity has a relatively high prevalence. A more clinically relevant bedside definition of paediatric shock is however urgently needed.

### Aetiology

Critically ill children such as children with shock, in our study and others, are often diagnosed with multiple underlying diseases [4, 6]. This study underlines that data is limited on the aetiology shock in African children, in part due to limited diagnostic capacity in low-resource settings which makes differentiation of underlying aetiologies challenging and based on clinical characteristics only [14].

Sepsis and septic shock are recognized as an important contributor to childhood mortality worldwide [15, 16]. In this study, children with shock diagnosed with sepsis as their main diagnosis indeed had the highest odds of death. Mortality in these children was higher than reported in Kenyan children with suspected septic shock (63% vs. 23%) [6]. Diagnosing sepsis is however challenging in most low-resource settings like Malawi due to limited microbiological testing. In our study site blood cultures are available and were performed in a third. Still sepsis was not commonly diagnosed (5.7%) and in an even smaller proportion a pathogen was isolated from blood cultures, although antibiotics were administered in almost 45% of children with shock. A possible explanation could be prior antibiotic administration which may have lowered prevalence of bacteraemia in our population. The organisms we found are in line with prior research from African settings [17, 18].

Gastroenteritis was the second diagnosis associated with high odds of death. Children with gastroenteritis were excluded from the FEAST trial, however Mbevi et al. reported that the majority of children with shock had diarrhoea [6]. Whether these children have gastroenteritis or gastrointestinal symptoms due to systemic illness is less clear, which makes choosing the appropriate treatment strategy challenging [19].

Respiratory disease was the most common diagnosis in this study, which included bronchiolitis, asthma and severe pneumonia, and these children had the lowest odd of dying. A similar proportion of children with shock were diagnosed with respiratory tract infection in the FEAST trial (42%) and by Mbevi et al (46%) [4, 6]. A potential explanation may be a pulmonary infection as the cause of circulatory failure. Alternatively, one could state that children with respiratory distress (and/or hypoxia) often have an increase in heart rate and respiratory rate and therefore may be “falsely” labelled as shocked applying the FEAST criteria. The low mortality in these children suggests that the latter may apply in our setting and possibly also in other studies [4].

### Limitations

Limitations of this study include the use of the main discharge or death diagnosis in a setting that allowed us to do some diagnostic testing, however, we did not perform comprehensive testing to assess all aetiologies of shock in all children. Furthermore, the use of routinely collected data from paper hospital admission files, including variables needed for inclusion criteria, contributed to the missing data. This might have resulted in an underestimation of prevalence and mortality, as previous studies have shown that files of children who died had more missing data, possibly due to prioritization of care over recording admission information [20]. Furthermore, not all admitted children could be included for further analysis of underlying aetiology, as parents or guardians did not give their consent or were discharged before consent (174 of 679 eligible children, 25.6%). This might have led to an overestimate of mortality in this group and an underestimate of diseases that could have led to faster recovery.

## Conclusion

Considering the high mortality, and relatively high prevalence, we conclude that shock is an important clinical presentation in Malawi. Our data underline that there is a need for a clinically relevant bedside definition for shock, not only to more precisely define the prevalence but more importantly to improve the clinical approach of shock in African children and facilitate research that can be easily compared. Our data suggest sepsis and gastroenteritis are common diseases associated with poor outcome. As aetiological data on shock in African children are limited and a comprehensive diagnostic study is lacking, these data contribute to our understanding of paediatric shock.

## Supporting information

S1 Table Definitions for shock used in the WHO ETAT guideline, FEAST trial and this study. (DOCX)

S2 Table Description of variables used in this study. (DOCX)

S3 Table Diagnostic categories of the included children (N = 505) and which main discharge diagnosis was considered for the diagnostic categories. (DOCX)

S4 Table Diagnostic categories in children who died (n = 75). (DOCX)

S1 Fig Case record form. (DOCX)

S1 Checklist Inclusivity in global research. (DOCX)

S2 Checklist STROBE statement—Checklist of items that should be included in reports of observational studies. (DOCX)
