# Supplementary material for: Prevalence, mortality, and aetiology of paediatric shock in a tertiary hospital in Malawi: A cohort study
Source: PLOS Glob Public Health. 2024 Jan 8;4(1):e0002282. doi: 10.1371/journal.pgph.0002282 (PMC10773928; doi:10.1371/journal.pgph.0002282)
Supplement: S1 Table — (DOCX) [file pgph.0002282.s001.docx]

**S1 Table: Definitions for shock used in the WHO ETAT guideline, FEAST trial and this study**

| **Guideline or study** | **Definition or inclusion/exclusion criteria** |
| --- | --- |
| WHO ETAT^1^: shock | Cold extremities *with* capillary refill > 3 s *and* a weak, fast pulse (all signs must be present) |
| WHO ETAT^1^: (severely) impaired circulation | Cold extremities *or* a weak and fast pulse *or* capillary refill > 3 s |
| FEAST^2^ | “Children were eligible for inclusion in the study if they were between 60 days and 12 years of age and presented with a severe febrile illness complicated by impaired consciousness (prostration or coma), respiratory distress (increased work of breathing), or both, and with impaired perfusion, as evidenced by one or more of the following: a capillary refill time of 3 or more seconds, lower-limb temperature gradient, weak radial-pulse volume, or severe tachycardia (>180 beats per minute in children younger than 12 months of age, >160 beats per minute in children 1 to 5 years of age, or >140 beats per minute in children 5 years or older). Exclusion criteria were severe malnutrition, gastroenteritis, non-infectious causes of shock (e.g., trauma, surgery, or burns), and conditions for which volume expansion is contraindicated.” |
| Modified FEAST (this study) | Children aged 2 months to 16 years with impaired  consciousness (lethargy or Blantyre Coma Score (BCS) <5), respiratory distress (increased work of breathing, or both, *and* at least one sign or impaired circulation: capillary refill time > 3 seconds, lower-limb temperature gradient/cold peripheries, weak radial pulse, severe tachycardia (>180 bpm if <12 months, >160 bpm if 1-5 years of age, 140 bpm if 5-12 years of age, >120 bpm if 12-16 years of age). |

1. Updated guideline: paediatric emergency triage, assessment and treatment. Geneva: World Health Organization,; 2016.

2. Maitland K, Kiguli S, Opoka RO, Engoru C, Olupot-Olupot P, Akech SO, et al. Mortality after fluid bolus in African children with severe infection. N Engl J Med. 2011;364(26):2483-95.
