# Supplementary material for: Prevalence, mortality, and aetiology of paediatric shock in a tertiary hospital in Malawi: A cohort study
Source: PLOS Glob Public Health. 2024 Jan 8;4(1):e0002282. doi: 10.1371/journal.pgph.0002282 (PMC10773928; doi:10.1371/journal.pgph.0002282)
Supplement: S2 Table — (DOCX) [file pgph.0002282.s002.docx]

**S2 Table: Description of variables used in this study**

| **Variable** | **Details** |
| --- | --- |
| Packed cell volume (PCV) | Routinely sampled in children with fever or if indicated by the clinician. |
| Malaria test | Microscopy slide or rapid test. Routinely sampled in children with fever or if indicated by the clinician |
| HIV testing | Offered to all admitted children |
| Blood gas | Not routinely available |
| Suspected sepsis | Based on the interpretation of the potential diagnosis by the attending clinician, and if considered a blood gas was taken |
| Anaemia | Haemoglobin <10g/dl or PCV<30% |
| Severe anaemia | Haemoglobin ≤5g/dL or PCV≤15% |
| Hypoglycaemia | Defined as <2.4 or <3.0 mmol/L in well/fairly nourished or severely malnourished patients respectively |
