# Supplementary material for: Prevalence, mortality, and aetiology of paediatric shock in a tertiary hospital in Malawi: A cohort study
Source: PLOS Glob Public Health. 2024 Jan 8;4(1):e0002282. doi: 10.1371/journal.pgph.0002282 (PMC10773928; doi:10.1371/journal.pgph.0002282)
Supplement: S3 Table — (DOCX) [file pgph.0002282.s003.docx]

**S3 Table: Diagnostic categories of the included children (N=505) and which main discharge diagnosis was considered for the diagnostic categories**

| **Diagnostic category** | **Main discharge diagnoses included in the diagnostic category** |
| --- | --- |
| Reactive airway disease | Bronchiolitis, asthma, viral induced wheeze |
| Severe pneumonia | Pneumonia, tuberculosis, empyema, pneumocystis carinii pneumonia |
| Gastroenteritis | Acute gastroenteritis, chronic gastroenteritis, severe acute malnutrition |
| Sepsis | Sepsis, disseminated staphylococcal disease, urinary tract infection |
| Cardiac disease | Rheumatic heart disease, congestive heart failure, acute rheumatic fever, myocarditis, dilated cardiomyopathy, atrial fibrillation, cardiogenic shock |
| Malaria | Cerebral malaria, severe malaria |
| Neurological disease | Meningitis, rabies, encephalitis, brain tumor, epilepsy, epilepsy, cerebral palsy, febrile convulsions |
| Other | All other main diagnoses |
