# Supplementary material for: Prevalence, mortality, and aetiology of paediatric shock in a tertiary hospital in Malawi: A cohort study
Source: PLOS Glob Public Health. 2024 Jan 8;4(1):e0002282. doi: 10.1371/journal.pgph.0002282 (PMC10773928; doi:10.1371/journal.pgph.0002282)
Supplement: S4 Table — (DOCX) [file pgph.0002282.s004.docx]

**S4 Table: Diagnostic categories in children who died (N=75)**

| Diagnostic category | n (%) |
| --- | --- |
| Gastro-enteritis | 21 (28.0) |
| Sepsis | 17 (22.7) |
| Malaria | 12 (16.0) |
| Cardiac disease | 7 (9.3) |
| Neurological disease | 7 (9.3) |
| Severe pneumonia | 2 (2.7) |
| Viral/reactive respiratory diseases | 0 (-) |
| Other | 9 (12.0) |
