# Supplementary material for: Prevalence, mortality, and aetiology of paediatric shock in a tertiary hospital in Malawi: A cohort study
Source: PLOS Glob Public Health. 2024 Jan 8;4(1):e0002282. doi: 10.1371/journal.pgph.0002282 (PMC10773928; doi:10.1371/journal.pgph.0002282)
Supplement: S1 Fig — (DOCX) [file pgph.0002282.s005.docx]

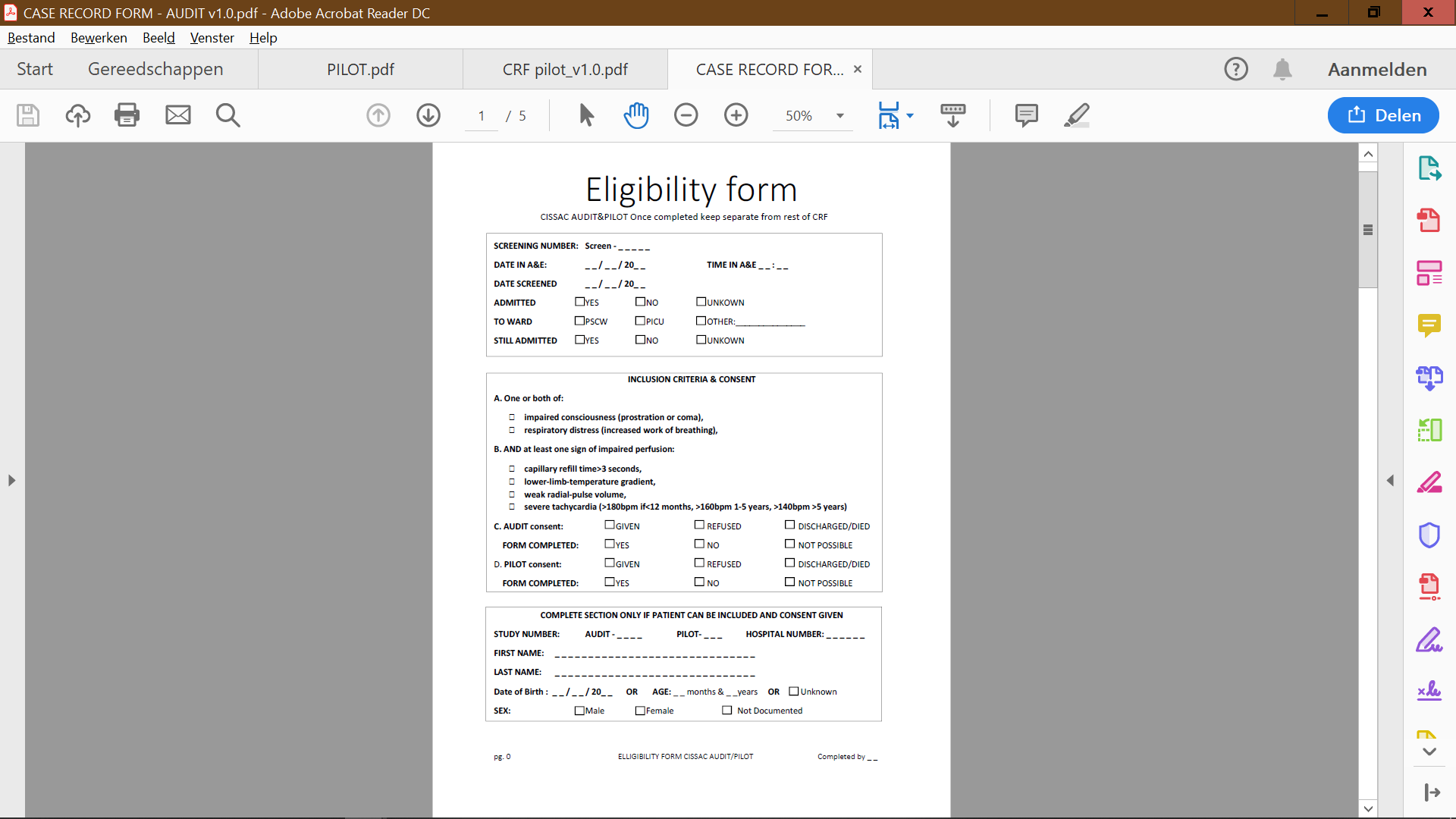


**S1 Fig: Case Record Form (page 1/5)**


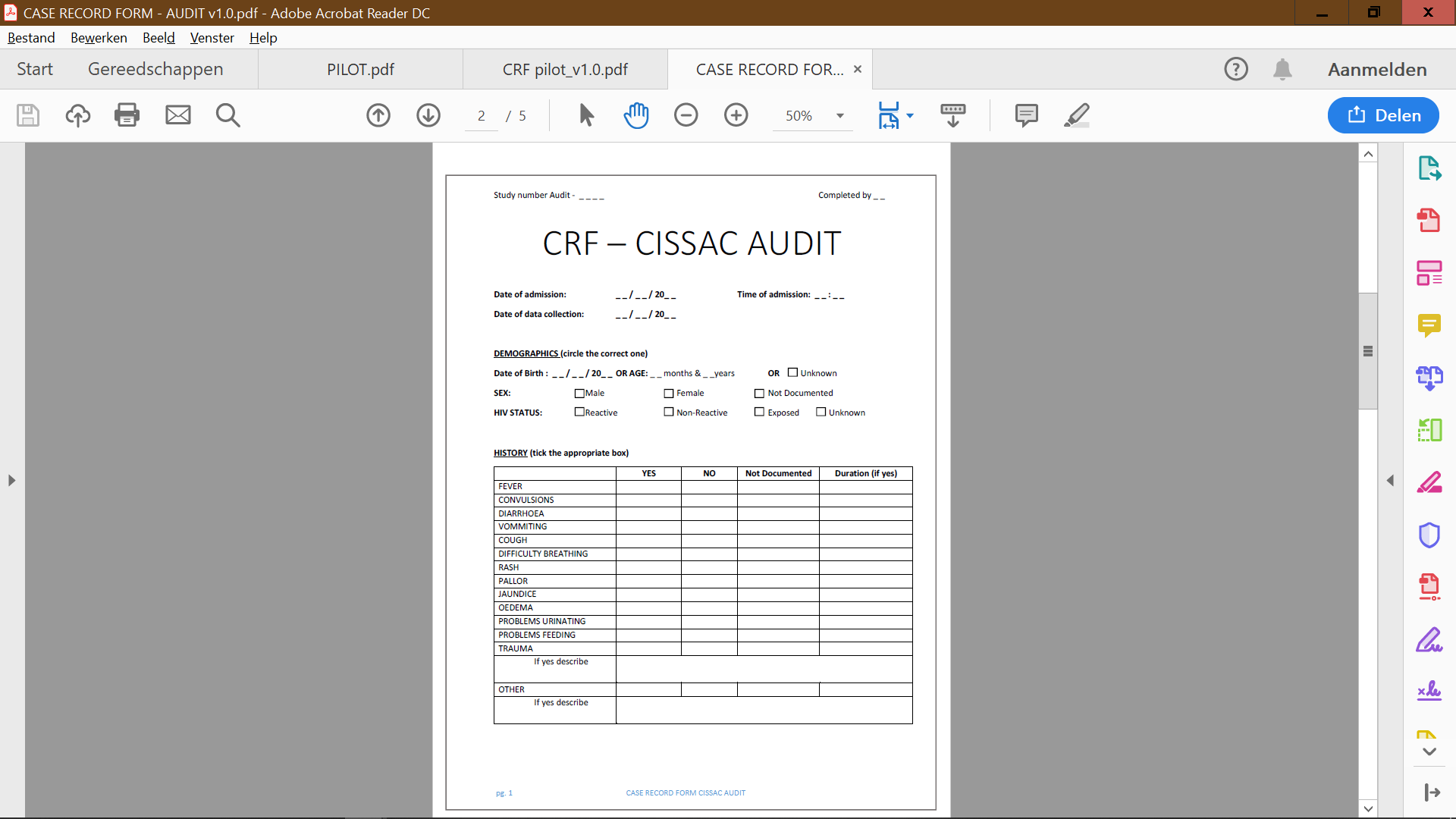


**S1 Fig: Case Record Form (page 2/5)**

**
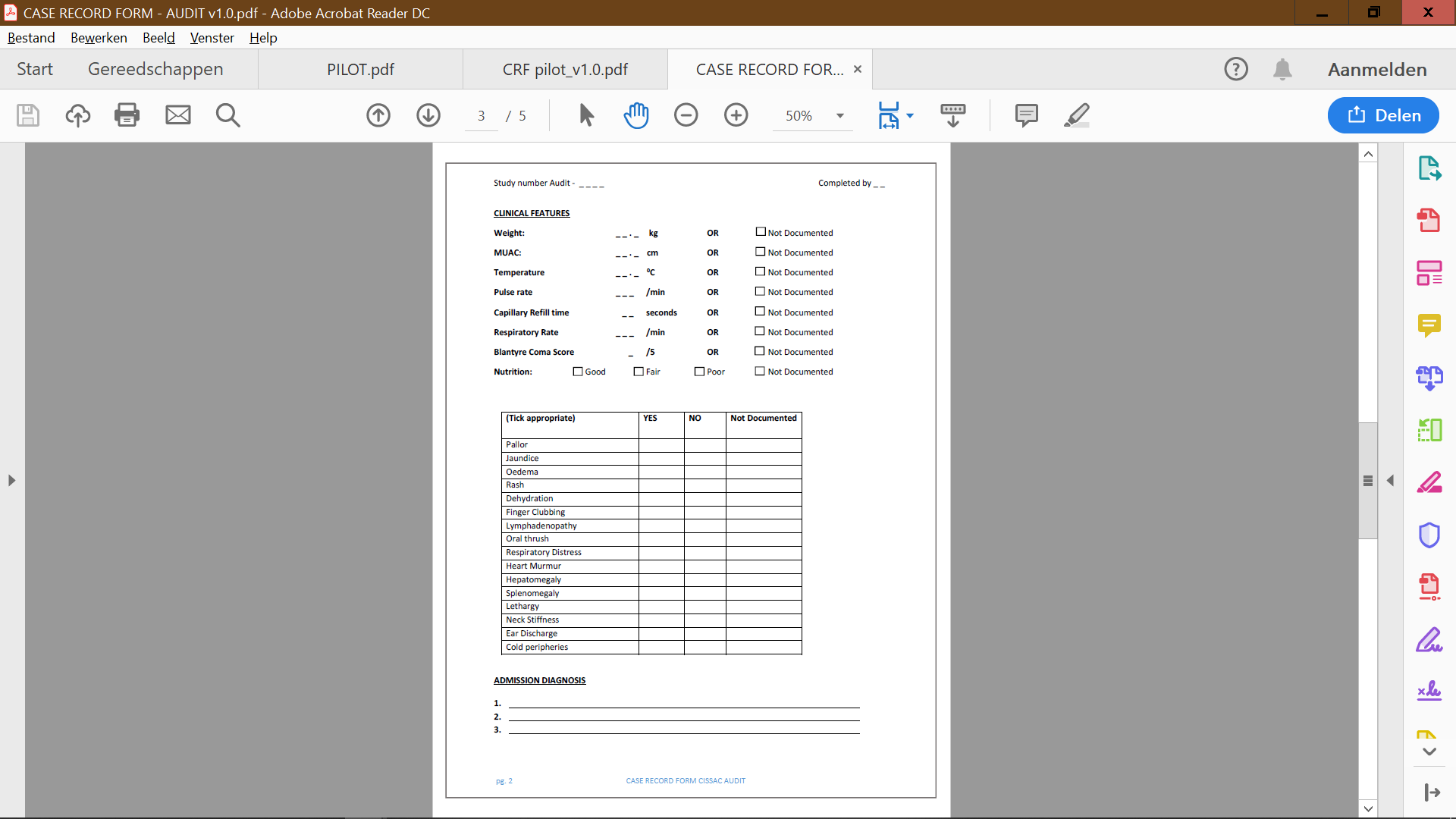
**

**S1 Fig: Case Record Form (page 3/5)**


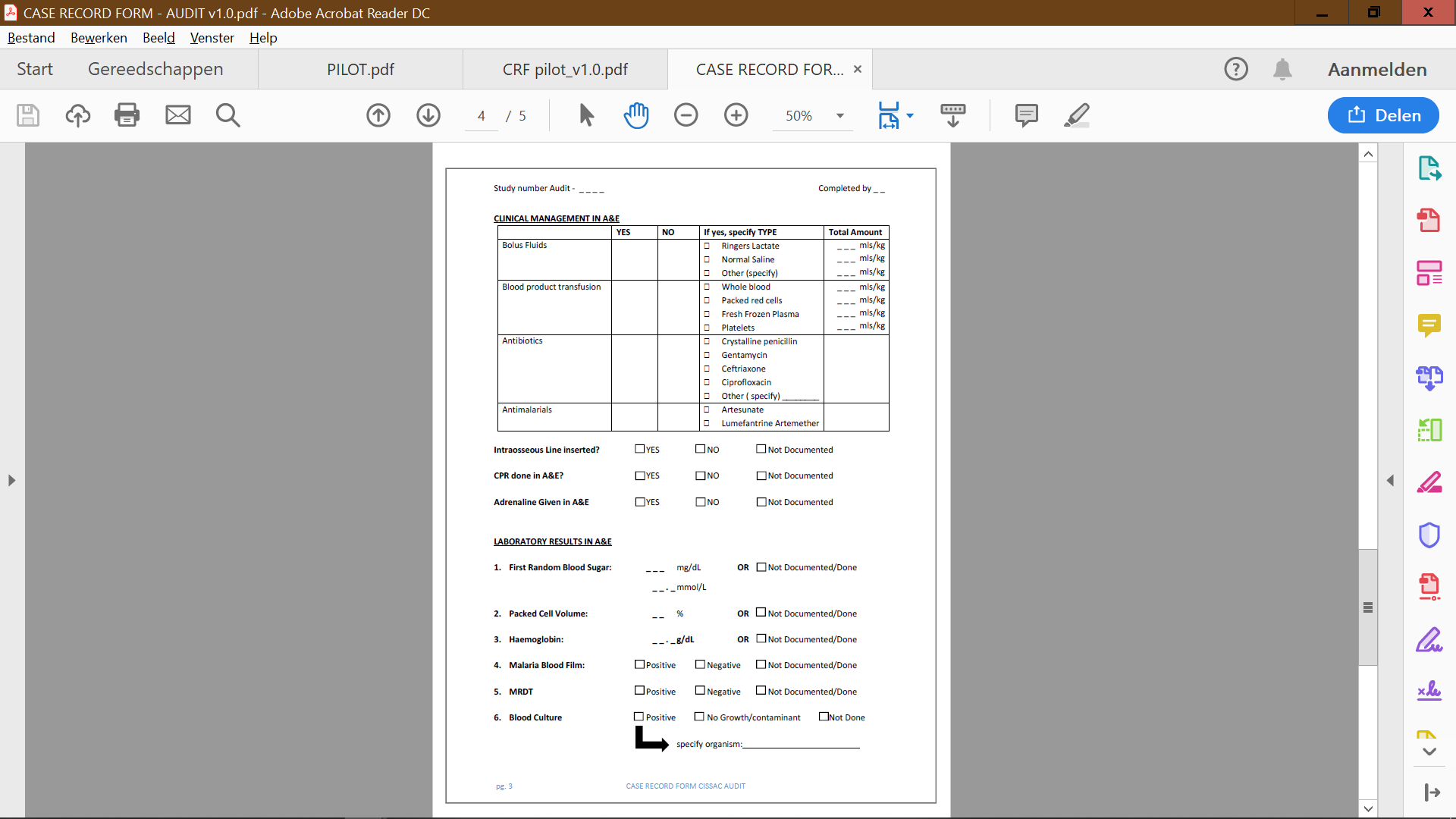


**S1 Fig: Case Record Form (page 4/5)**


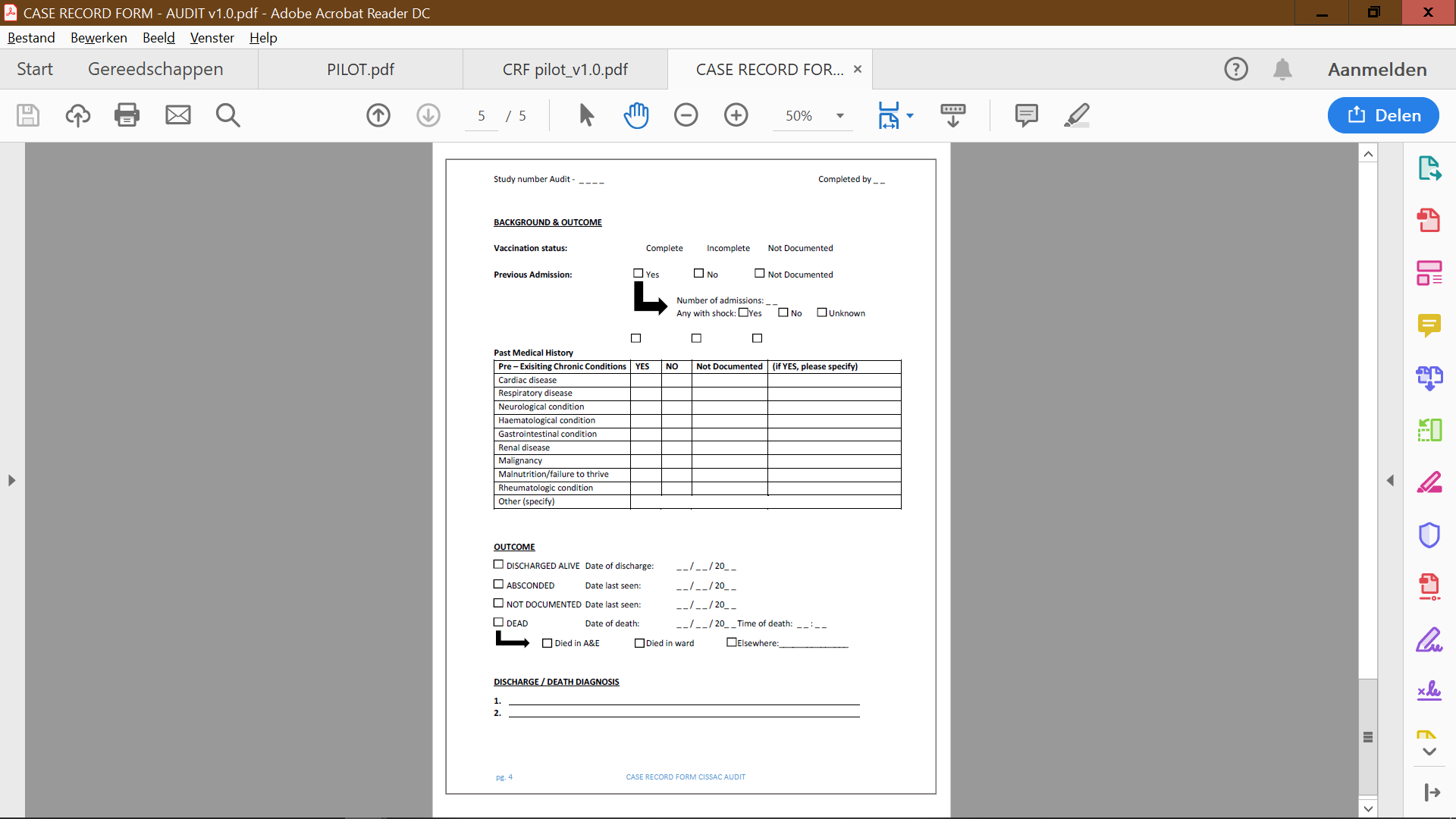


**S1 Fig: Case Record Form (page 5/5)**
